# Supplementary material for: The genome sequence of Quercus petraea Liebl., 1784 (Fagales: Fagaceae)
Source: Wellcome Open Res. 2026 Jul 29;11:483. [Version 1] doi: 10.12688/wellcomeopenres.27285.1 (PMC13560820; doi:10.12688/wellcomeopenres.27285.1)
Supplement: Peer review history

# Reviewer report: Reviewer response for version 1

**DOI:** 10.21956/wellcomeopenres.30029.r163153

**Published:** 2026-08-21

**Authors:** Kapeel Chougule

**Affiliations:** 1 Cold Spring Harbor Laboratory, New York, USA

The authors present a high-quality haplotype-resolved genome assembly of *Quercus petraea* generated as part of the Darwin Tree of Life project. PacBio HiFi and Hi-C sequencing produced two haplotypes, with 99.41% of haplotype 1 assigned to 12 chromosomal pseudomolecules. The assembly has high consensus accuracy and completeness (QV 63.5 and 99.13% combined k-mer completeness) and provides a useful genomic resource.

 The sequencing, assembly and quality-assessment approaches are appropriate, I have only a few points that would improve clarity:

**1.** The authors report genome-size estimates of 855.19 Mb from GenomeScope2 and 990 Mb from flow cytometry. It should be explicitly stated which estimate was used to calculate the reported ~65× sequencing coverage, and the difference between the two genome-size estimates could be briefly discussed.

**2.** The authors state that scaffold order/orientation remains uncertain in regions of chromosomes 3 (~33.10–36.19 Mb), 6 (~0.82–9.50 Mb), and 9 (~28.90–30.78 Mb). It would be useful to briefly explain the source of these uncertainties (e.g., repetitive sequence or ambiguous Hi-C support), as this information is relevant to downstream users of the reference assembly.

**3.** Haplotype 1 was assembled to chromosome level whereas haplotype 2 remains at scaffold level, despite broadly comparable assembly statistics. Please clarify whether this reflects the standard DToL assembly/curation strategy or a limitation of the available scaffolding/phasing information.

Are sufficient details of methods and materials provided to allow replication by others?

Yes

Is the rationale for creating the dataset(s) clearly described?

Yes

Are the datasets clearly presented in a useable and accessible format?

Yes

Are the protocols appropriate and is the work technically sound?

Yes

Reviewer Expertise:

plant genomics

I confirm that I have read this submission and believe that I have an appropriate level of expertise to confirm that it is of an acceptable scientific standard.

# Reviewer report: Reviewer response for version 1

**DOI:** 10.21956/wellcomeopenres.30029.r163152

**Published:** 2026-08-18

**Authors:** Peng-Fei Ma

**Affiliations:** 1 Chinese Academy of Sciences, Kunming, China

In this study, the genome of *Quercus petraea* (sessile oak) was sequenced and assembled. As one of the two native oak species in the UK, it is ecologically significant. The assembly was resolved at the haplotype-level with most of haplotype 1 sequences scaffolded into 12 chromosomal pseudomolecules. The assembly quality is high with an estimated QV of 63.5 and 99.13% k-mer completeness. In addition, a total of 32,253 protein-coding genes were annotated. In short, the improved reference genome of Quercus petraea by the Darwin Tree of Life project here will be helpful for the conservation and management of oak populations and associated biodiversity in the UK.

Are sufficient details of methods and materials provided to allow replication by others?

Yes

Is the rationale for creating the dataset(s) clearly described?

Yes

Are the datasets clearly presented in a useable and accessible format?

Yes

Are the protocols appropriate and is the work technically sound?

Yes

Reviewer Expertise:

Plant genomics and evolution

I confirm that I have read this submission and believe that I have an appropriate level of expertise to confirm that it is of an acceptable scientific standard.

# Reviewer report: Reviewer response for version 1

**DOI:** 10.21956/wellcomeopenres.30029.r163154

**Published:** 2026-08-10

**Authors:** Nils Stein

**Affiliations:** 1 Leibniz Institute of Plant Genetics and Crop Plant Research (IPK), Seeland, Germany

The authors present a high quality chromosome scale assembly of the genome of Quercus petrea, a native tree species of the UK. The genome was sequenced as part of the DToL project taking advantage of the standardized protocols and procedures.

 genome size was estimated by GenomeScop2 and flow cytometry giving estimates that differ by 10%. Sequence coverage was determined based on the kmer based estimation, however, this is not explicitly clear from the report.

 Chromosomes 6 and 9 show strong interaction - is this artificial? is this related to the NOR region? Should be commented to clarify that these patterns do not point to artifacts.

Are sufficient details of methods and materials provided to allow replication by others?

Yes

Is the rationale for creating the dataset(s) clearly described?

Yes

Are the datasets clearly presented in a useable and accessible format?

Yes

Are the protocols appropriate and is the work technically sound?

Yes

Reviewer Expertise:

Plant genetics / genomics

I confirm that I have read this submission and believe that I have an appropriate level of expertise to confirm that it is of an acceptable scientific standard.
